# MS-Based Analytical Techniques: Advances in Spray-Based Methods and EI-LC-MS Applications

**DOI:** 10.1155/2018/1308167

**Published:** 2018-04-23

**Authors:** Federica Bianchi, Nicolò Riboni, Veronica Termopoli, Lucia Mendez, Isabel Medina, Leopold Ilag, Achille Cappiello, Maria Careri

**Affiliations:** ^1^Department of Chemistry, Life Sciences, and Environmental Sustainability, University of Parma, Parco Area delle Scienze 17/A, 43124 Parma, Italy; ^2^Department of Environmental Science and Analytical Chemistry, Stockholm University, 10691 Stockholm, Sweden; ^3^Department of Pure and Applied Sciences, LC-MS Laboratory, Piazza Rinascimento 6, 61029 Urbino, Italy; ^4^Instituto de Investigaciones Marinas, Spanish National Research Council (IIM-CSIC), Eduardo Cabello 6, 36208 Vigo, Spain

## Abstract

Mass spectrometry is the most powerful technique for the detection and identification of organic compounds. It can provide molecular weight information and a wealth of structural details that give a unique fingerprint for each analyte. Due to these characteristics, mass spectrometry-based analytical methods are showing an increasing interest in the scientific community, especially in food safety, environmental, and forensic investigation areas where the simultaneous detection of targeted and nontargeted compounds represents a key factor. In addition, safety risks can be identified at the early stage through online and real-time analytical methodologies. In this context, several efforts have been made to achieve analytical instrumentation able to perform real-time analysis in the native environment of samples and to generate highly informative spectra. This review article provides a survey of some instrumental innovations and their applications with particular attention to spray-based MS methods and food analysis issues. The survey will attempt to cover the state of the art from 2012 up to 2017.

## 1. Introduction

Mass spectrometry (MS) is one of the most powerful techniques for the detection and identification of organic and inorganic compounds. Being able to provide both molecular weight and structural information [1], it is widely used in analytical laboratories for academic research, industrial product development, and regulatory compliance as well as for proteomic or metabolomic studies, DNA characterization, drug discovery, environmental monitoring, food analysis, forensics, and homeland security.

A plethora of analytical MS-based methods based on the use of both stand-alone instruments and mass spectrometers coupled to different separation techniques such as gas and liquid chromatography (GC and LC) or capillary electrophoresis (CE) have been developed and validated in order to analyze complex matrices. Interesting review articles and book chapters dealing with advances in ionization for mass spectrometry have been lately published [2–9].

Recently, the advent of ambient MS technology paved the way for the development of a great variety of applications and innovations characterized by high throughput: the challenge of analyzing samples in their native state without sample treatment encouraged the development of new techniques among which are the spray-based ionization ones including desorption electrospray ionization (DESI) [10], paper spray ionization (PSI) [11], laser ablation electrospray ionization (LAESI) [12], and easy ambient sonic-spray ionization (EASI) [13].

Novel materials and new instrumental configurations are under study to enhance the performance of the different ion sources. Safety risks can be identified at the early stages through nontargeted monitoring technologies. Furthermore, the variety of fragmentation strategies that can be combined in new instrumentation overall enhances work in the omics fields, particularly proteomics and metabolomics.

Although MS-based methods are getting progressively more powerful, reliable, and easily available, the main drawbacks are still related to sample complexity and preparation, mass accuracy, often requiring the use of high-resolution mass spectrometry (HRMS) to guarantee the univocal identification of the targeted compounds, and the need of high-throughput and screening analyses when a great number of samples have to be analyzed.

The aim of the proposed special issue is to cover the aspects regarding emerging features of MS-based techniques focusing on innovative LC-MS studies and ambient MS with particular attention to the spray-based ionization techniques. New materials, prototypes, and instrumental configurations able to increase the performance of the developed methods will be presented and discussed. Finally, an overview of the most recent MS-based methods in food analysis will be given. This survey will attempt to cover the state of the art from 2012 up to 2017.

## 2. Advances in LC-MS

Electrospray ionization (ESI) is the technique of choice to produce ions suitable for mass analysis. ESI spectra typically are characterized by single protonated or deprotonated molecular ion (M + H)^+^, (M − H)^−^, and/or adduct ions. The low fragmentation is a limitation in compound characterization through the use of reliable electronic libraries, making necessary the use of multistage MS (MS/MS, MS^*n*^) or HRMS to compensate the limited structural information. LC-ESI with triple quadrupole (QqQ) MS is the most used technique for qualitative and quantitative determination of targeted nonvolatile compounds in forensic and food applications [14, 15]. In 2016, Remane et al. reviewed the literature on applications of LC-MS/MS in clinical, forensic toxicology, and doping control since 2006 [16]. It must be noted that ESI response is strictly affected by the mobile phase and sample composition as well as by the presence of coeluting interfering compounds, which may interfere with the ionization process. These phenomena are known as “matrix effects” (MEs) and can alter the response of the analytes causing either signal suppression or enhancement [17, 18]. The occurrence of ME introduces some critical analytical shortcomings in quantitative analysis by LC-MS such as reduced sensitivity, nonlinear response, and low precision. In addition, the physicochemical properties of the analytes can play an issue in the instrument response, thus introducing additional limitations. The combination of powerful MS detectors with LC has solved many problems in structural elucidation of unknown hazardous compounds [19]. In this context, HRMS is capable of providing full spectral information by adding high mass resolving power and accuracy to achieve selectivity and capability for accurate mass measurements [20–22]. HRMS is characterized by higher mass resolution, defined as the mass difference between two mass spectral peaks that can be clearly distinguished [23], and higher mass accuracy (even better than 1 ppm). In addition, high mass resolving power allows discrimination between isobaric interference and ion of interest, leading to an accurate mass measurement even with a complex background. These features increase MS selectivity for the screening of nontargeted compounds in complex matrices, providing a list of possible elemental compositions. Fu et al. have shown how important and efficient is the use of nontargeted screening with LC-HRMS to ensure quality and safety of food [24], whereas Mattarozzi and coworkers exploited the capability of HRMS for the rapid determination of melamine from melamine tableware [25]. On the downside, HRMS-based methods generate complicated data that must be processed for “total ion fragment spectra” to obtain high-quality mass spectral information. Moreover, the mass of protonated or deprotonated molecules is not sufficient to prevent unambiguous compound identification. Hence, the use of spectra-matching approaches that utilize fragmentation ions could be added to achieve additional information on the detected compounds [26, 27]. False positives and false negatives are the major obstacles when screening complex samples. False negatives can occur due to very low concentrations, matrix interferences and suppression, and weak or no ionization. Due to these disadvantages, the practical application of LC-MS and LC-HRMS is still far from the immediacy and simplicity of GC-MS. Taking into account that electron ionization (EI) allows us to obtain characteristic and highly reproducible fragmentation of the analytes, a considerable effort has been devoted by the scientific community to increase compatibility between LC and EI-based MS to develop reliable, easy-to-use, and flawless interfaces. Moreover, implementation of EI fragmentation to LC-amenable compounds could pave the way for many new fields of research.

Recent developments in miniaturized mass spectrometers have enabled these developments to be carried out to portable on-scene detection. In the next paragraphs, some of the most popular and promising techniques are described: among them are LC-MS based on EI interfaces and spray-based ionization techniques.

### 2.1. LC-MS Based on Electron Ionization-Mass Spectrometry Interfaces

It is known that EI is ideal for the detection of a large number of GC-amenable compounds, but with an appropriate combination of several measures, it can become suitable for many LC-amenable small molecules having MW up to approx. 600 Da. These compounds can be efficiently converted into the gas phase, fast enough to be ionized before any decomposition process.

For analysis of small-to-medium molecules, the coupling between LC and EI-MS represents a valid strategy for overcoming the main disadvantages related to ESI ionization and the use of costly and complicated techniques involving HRMS instrumentations. Furthermore, EI provides a rich fragmentation pattern with a significant amount of structural information allowing a unique automated identification with structures at the isomer level [28]. Hence, the ability of EI for tentative identification of GC-amenable compounds is unparalleled even without HRMS.

On the downside, the coupling between LC and MS based on EI represents a significant issue in the field of analytical chemistry. The reason may be explained by the antagonist conditions of operating. The first one typically works at ambient temperature and uses very high pressure for the efficient separation of the analytes, which are sometimes dissolved in a complex mobile phase. The second one operates at a very high vacuum and high temperature. Therefore, the effort of achieving and maintaining the high vacuum required for mass spectrometry is in contrast with the intrinsic nature of HPLC, predominantly operating at high solvent flow rates. Also, the low tolerance of mass spectrometers for nonvolatile mobile phase components contrasts with an HPLC dependence on nonvolatile buffers to achieve high-resolution separations.

Since the year 2000, a few groups of researchers are working on the development of an efficient EI-based LC-MS interface.

**Figure 1:**
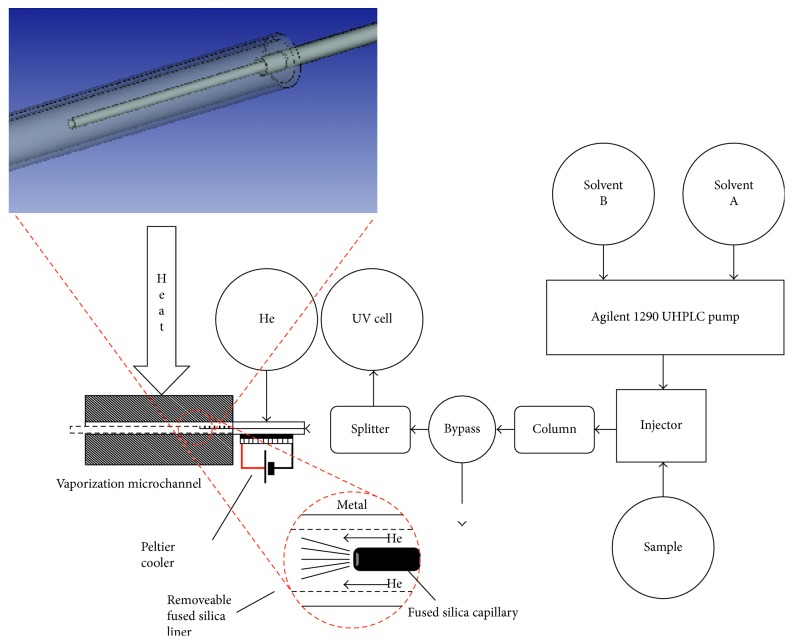
Global layout of the fully assembled system; the LEI interface, in gray, is between the UHPLC system and the MS detector. In the red circle, the vaporization zone is highlighted. Reprinted with permission from [33].

Cappiello and his group played a significant role in the innovation and improvement of LC-EI-MS interfacing and designing a series of systems characterized by steadily increasing performance. Firstly, they presented a prototype of the LC-MS interface called Direct-EI [29–31] based on direct coupling of a low flow rate nano-HPLC with a high-vacuum EI source. The interface governs the direct introduction of a liquid-phase sample into the EI source of the mass spectrometer and the complete conversion of the liquid effluent to the gas phase prior to a conventional electron-assisted ionization. The core of the interface is represented by the nebulizer, which consists of a fused silica/PEEK capillary, to guarantee a sufficient thermal insulation. This interface was used in many different applications, not only in combination with chromatography but also in direct analysis, as a universal detector for the targeted compound. However, both nebulization and vaporization take place inside the ion source, leading to some drawbacks linked to capillary blockings. This concern is mainly due to premature evaporation of the solvent making the analysis very difficult under routine conditions. To meet the challenges of analyzing nontargeted compounds exploiting full potential of EI and the quantification of target compounds at low concentration in complex matrices, Termopoli et al. presented a new, robust, efficient interfacing mechanism coming from the ground up [32]. The new interface is called “liquid-EI” (LEI). The interface is completely independent from the rest of the instrumentation and can be adapted to any gas chromatography-mass spectrometry system, as an add-on for a rapid LC-MS conversion. Secondly, with some little tricks, it can be used with any HPLC system. Nanopumps and capillary pumps allow direct connection, and conventional HPLC needs the use of a two-way splitter to reduce the column flow rate to a level that is compatible with the interface, which is normally between 0.5 and 1 *µ*l/min. In an LEI interface, the vaporization of the LC eluate is carried out at atmospheric pressure inside a suitable, independent microchannel right before entering the ion source, called the vaporization microchannel, representing the core of the interface. It is designed to uncouple and separate the atmospheric pressure found at the end of the HPLC system with the high-vacuum zone of the ion source. This specific place is narrow enough to prevent vacuum from entering into the spray region allowing us to have an atmospheric pressure zone where the vaporization process takes place. A removable silica-deactivated liner ensures a perfect conversion into a gas phase before entering the mass spectrometer. A narrower fused silica capillary, called the inlet, penetrates in the first portion of the liner and releases the LC eluate. An inert gas flow surrounds the gas phase through the vaporization microchannel and helps high boiling compounds to vaporize.  Figure 1 shows a complete layout of the LC-MS system equipped with the LEI interface.

The rapid vaporization offered by the lined microchannel reduces the chance of thermal decomposition and capillary blockings, broadening the range of suitable applications, especially those regarding nontargeted analytes. Remarkable results were achieved in different conditions and applications.

Over the past years, Seemann and his group developed the supersonic molecular beams (SMB) LC-MS interface [34]. Their studies started from the knowledge that standard emission energy (70 eV) used in EI is not ideal for many NIST library compounds that have a weak (below 2% relative abundance) or no molecular ion. This issue is a critical point when very large and thermally labile compounds are analyzed. Furthermore, these analytes are usually less volatile and require higher EI ion source temperatures with further intra-ion source degradation, resulting in weaker molecular ion production. To achieve a reliable EI-based sample identification, a more intense production of molecular ion is needed. Thus, the best ionization method should provide the informative library searchable EI fragments combined with enhanced molecular ions, especially for the compounds that are not included in the commercially available EI libraries. Taking into account these considerations, they present a novel concept of the LC-SMB-MS system, based on the use of supersonic molecular beams, as a medium for electron ionization of vibrationally cold sample molecules in a fly-through ion source. It is able to generate library searchable EI spectra and a more intense molecular ion.

**Figure 2:**
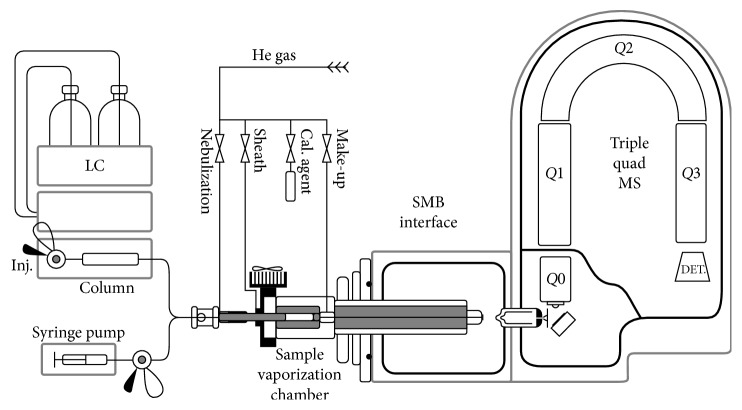
EI-LC-MS with the SMB system outline. The liquid is introduced either from the HPLC system after its column or from a syringe pump to the heated vaporization chamber through a pneumatic nebulizer. The helium nebulization gas enters the SMB interface through a nebulization gas line, sheath gas line, and nozzle make-up gas line. Reprinted with permission from [35].

The LC-SMB-MS apparatus is schematically shown in Figure 2.

A thorough evaluation of the interface, comprising identification of unknown compounds using obtained library searchable EI mass spectra, enhanced production of molecular ions, demonstration of the absence of matrix effects, simultaneous determination of semipolar and nonpolar compounds with reasonable detection limit, and low-cost instrumentation, was provided by that research group. The group of Seemann demonstrates the feasibility of the SMB interface as a valid tool in the analysis of unknown compounds and as a low-cost LC-EI-MS system.

A third group of researchers, headed by Rigano, presented a new nano-LC-EI-MS for the determination of free fatty acids (FFAs) in mussels [34]. A selective and sensitive nano-LC-EI-MS analytical method to investigate the FFA profile in marine organisms and to monitor marine sentinels for the assessment of environmental pollution effects was developed [35]. FFAs are minor components of the lipidome, and they are usually analyzed by GC after a derivatization step, such as methylation or trimethylsilylation, is performed to convert FA into less-polar and more-volatile moieties and improve their separation [9, 36]. However, the derivatization step, if not properly selected, can modify the FA profile due to nonhomogeneous derivatization efficiency among different compounds (saturated, unsaturated, and polyunsaturated fatty acids). In addition, oxidation or isomerization products can be generated. Relative to this issue, LC can benefit over GC techniques from direct injection of FFAs in their intact form, without any pretreatment. On the other hand, direct coupling with EI-MS can benefit from the highly informative, repeatable, and reliable MS fragmentation. Drawing conclusions of the several attempts made by each group, they are following a distinctive pathway to obtain a common goal, the development of a more useful and universal LC-EI-MS interface.

Regarding Direct-EI LC-MS, recent studies have been carried out also to increase the inertness of the electron ionization ion source by developing new materials [37, 38].

As already stated, the vaporization surface of an electron ionization MS source is a key parameter for the detection and characterization of targeted and untargeted analytes: it is known that difficulties in the vaporization process arise when compounds characterized by high molecular weight and/or polarity have to be analyzed, thus requiring both the use of inert ion sources to reduce the interactions of the analytes with the stainless steel ion source and the use of high source temperatures to promote analyte vaporization. In this field, Magrini et al. [37] proved that the use of a commercially available ceramic coating is able to improve the detection of high molecular weight and high boiling compounds like polycyclic aromatic hydrocarbons (PAHs) and hormones. More recently, Riboni et al. [38] were able to increase the inertness of the electron ionization ion source by developing different sol-gel coatings based on silica, titania, and zirconia. Again, the developed coatings were tested for the Direct-EI LC-MS determination of PAHs and steroids. The best performances in terms of both signal peak intensity and peak width were obtained by using the silica-based coating, obtaining detection limits in the low ng/ml range with a good precision (RSD <9% for PAHs and <11% for hormones). No problems associated to ion cleaning were observed after prolonged use.

## 3. Advances in Spray-Based Ionization Techniques

Nowadays, there is also a growing interest in the development of real-time analytical technologies capable of allowing the direct detection of trace analytes in complex samples, especially in their native environment. The development of a new class of techniques, better known as “ambient ionization techniques,” has introduced a revolution in the ionization field. These techniques are able to generate ions directly from native environment of the sample at ambient pressure, without any tedious sample preparation steps or laborious time-consuming chromatographic separation.

Technically, spray-based ionization techniques are based on the use of electrospray droplets to extract the analytes from the sample and transfer them to the mass spectrometer. The most common spray-based ionization technique is DESI, in which a high-velocity pneumatically assisted ESI source generates charged microdroplets by the application of a proper potential on the ESI needle. The spray is directed towards the sample where the impact of the primary droplets with the substrate leads to the formation of a micrometer-sized thin solvent film, able to solubilize the analytes at the liquid-solid interface. Secondary droplets containing the analytes expelled by the film solvent are generated, and then, desolvation and ionization in the gas phase occur, as in the traditional ESI analysis. Finally, the ions are collected by the MS inlet.

In addition to DESI, several other techniques like nano-DESI, EASI, and LAESI have been proposed. Probe electrospray ionization (PESI) is another interesting approach based on the use of a solid conductive needle probe that replaces the traditional electrospray capillary for sample introduction. Similarly, PSI is a technique based on the loading of the sample onto a triangular piece of paper from which ions are generated by applying a high voltage in the presence of a proper solvent [11]. Spray-based methods are suitable for the analysis of different compounds, from small analytes, such as explosives [39–43], drugs [44–47], and food contaminants [48–50], to larger molecules such as lipids [51–53], peptides [54, 55], and proteins [56, 57].

### 3.1. Desorption Electrospray Ionization-Mass Spectrometry

DESI-MS is usually applied for surface desorption/ionization of analytes deposited on a probe material (PTFE, PMMA, glass, etc.) or directly from the sample surface. DESI-MS and DESI imaging have been successfully applied in different fields, such as forensic science [58], food control [59, 60], and clinical applications [61–63].

The derivatization of metabolites deposited in solution onto a glass plate by dropping the derivatizing reagent on the top of the dried analytes was proposed by Lubin et al. [64]. The authors successfully applied this technique to several samples, demonstrating the possibility of performing multiple and subsequent derivatization steps on the same spot.

**Figure 3:**
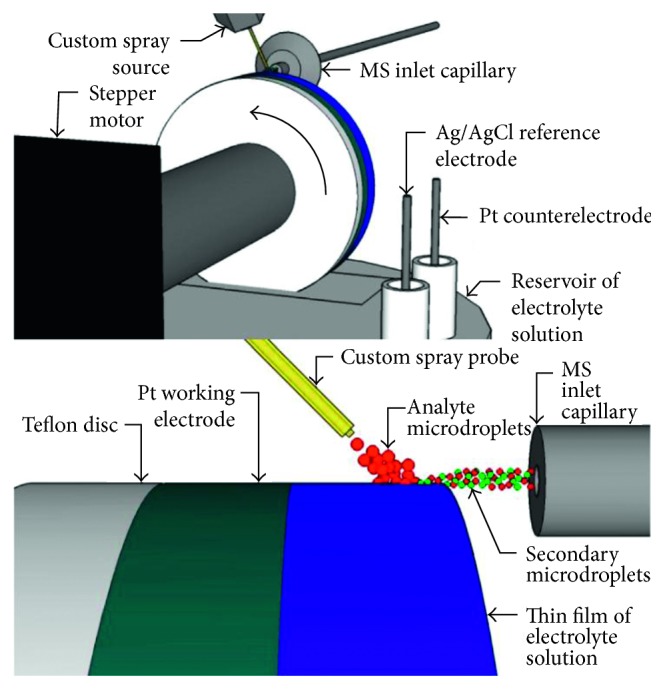
Schematic representation of the developed experimental setup. Reprinted with permission from [65].

An interesting approach was developed by Brown et al. [65] for the MS detection of fleeting reaction intermediates in electrochemical reactions utilizing a new *waterwheel* working electrode setup. The proposed technique allowed us to exploit DESI-MS operating at a low voltage. The new apparatus consisted of a round rotating platinum working electrode that was partially immersed in an aqueous electrolyte solution (Figure 3). During the rotation, a thin layer of liquid film was deposited on the electrode surface, as in a waterwheel. A three-electrode system was set by using a platinum wire counterelectrode and an Ag/AgCl reference electrode, immersed in the reservoir of electrolyte solution. The upper surface of the waterwheel was hit by a spray generated by a custom spray probe, thus allowing the formation of secondary droplets, analyte ionization, and their collection in the MS inlet. To avoid any electrochemical oxidation or reduction on the electrode surface, no high voltage was applied to the analyte spray, whereas a low potential (few volts) was applied to the platinum rotating electrode.

The authors tested the new apparatus towards the detection of a diimine intermediates during electrochemical oxidation of both uric acid and xanthine.

An MS-electrochemistry coupling was also proposed by Looi et al. [66], who developed a new online electrochemistry-liquid sample desorption electrospray ionization-mass spectrometry (EC-LS DESI-MS) system. In EC-LS DESI-MS, an electrosonic spray ionization source was used to generate a spray directed to the exit of the liquid sample capillary positioned perpendicularly to the spray and the MS inlet. Separately, a thin two-electrode flow-through EC cell was connected to a syringe pump and was used to perform oxidation/reduction processes. The ESSI-generated spray was able to impact the outer surface of the LS capillary, which is continuously coated by sample solution flowing at 200 *μ*l/h, thus allowing the ionization of the analytes. This prototype was developed and tested using *N*,*N*-dimethyl-*p*-phenylenediamine (DMPA). Although oxidation of DMPA was already observed as a result of ionization of DESI-MS in positive mode, by applying a proper voltage to the online electrochemical (EC) cell, it was possible to increase the yields of the oxidation products, thus improving method sensitivity.

Although DESI is usually coupled with high-resolution mass spectrometry, its coupling with LC is possible. A novel splitting method for LC-MS applications, which allows both very fast MS detection of analytes eluted from the LC column and their online collection, was presented by Cai et al. [67, 68]. In this approach, a PEEK capillary tube with a micro-orifice is used to couple DESI with the UPLC column. By using the proposed instrumental setup, a small amount of LC eluent (few nanoliters) is ionized by DESI with negligible time delay (6∼10 ms), whereas the remaining analytes exiting the tube outlet can be collected. In addition, online derivatization using reactive DESI is feasible increasing the charge of proteins and consequently enhancing the ionization yields.

**Figure 4:**
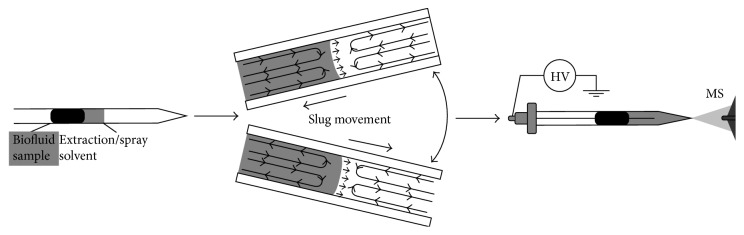
Schematic representation of the SFME-nano-ESI sample processing. Reprinted with permission from [69].

An interesting novel configuration has been recently developed by Ren et al. [69]. The authors developed a method coupling slug-flow microextraction (SFME) and nanoelectrospray ionization for the MS analysis of organic compounds in blood and urine. A disposable glass capillary with a pulled tip for nano-ESI was used to perform the entire extraction and ionization process (Figure 4). Two adjacent liquid plugs were formed by injecting 5 *µ*l of a proper organic solvent and 5 *µ*l of body fluid (urine or blood) into the capillary. Liquid-liquid extraction of the analytes was performed by both moving the capillary and applying a push-and-pull force through air pressure. After the extraction process, a high voltage is applied to the organic solvent plug to generate the nano-ESI for MS analysis.

The proposed method was tested for the extraction and detection of different analytes, namely, methamphetamine, benzoylecgonine, verapamil, amitriptyline, epitestosterone, 6-dehydrocholesterone, 5*α*-androstane-3*β*, 17*β*-diol-16-one, and stigmastadienone. Major analytical features were the reduced consumption of both the organic solvent and sample. The authors demonstrated that a direct derivatization of the extracted analytes in the organic phase was feasible, thus achieving excellent sensitivity with detection limits in the 0.03–0.8 ng/ml range.

Despite its name, nanospray DESI (nano-DESI) is based on a different instrumental configuration compared to the traditional DESI: its setup presents two different silica capillaries, one for solvent delivery and the other devoted to the formation of charged liquid spray in front of the MS inlet. The two capillaries are not in direct contact, thus producing a solvent bridge on the DESI surface. The second nanospray capillary produces a self-aspirating nanospray, which is generated by applying a high voltage between the MS inlet and the primary capillary. In comparison with DESI, nano-DESI is characterized by higher efficiency in liquid transportation and sampling performances.

The capabilities of nano-DESI-MS were tested for the determination of pollutants and organic components in atmospheric fine particles by Cain et al. [70], in environmental aerosol by Tao et al. [71], and in clouds by Boone et al. [72]. In the clinical and pharmaceutical fields, both nano-DESI-MS and nano-DESI-MS imaging proved to be excellent techniques for the analysis of pharmaceuticals, biomolecules, and metabolites [73–77].

**Figure 5:**
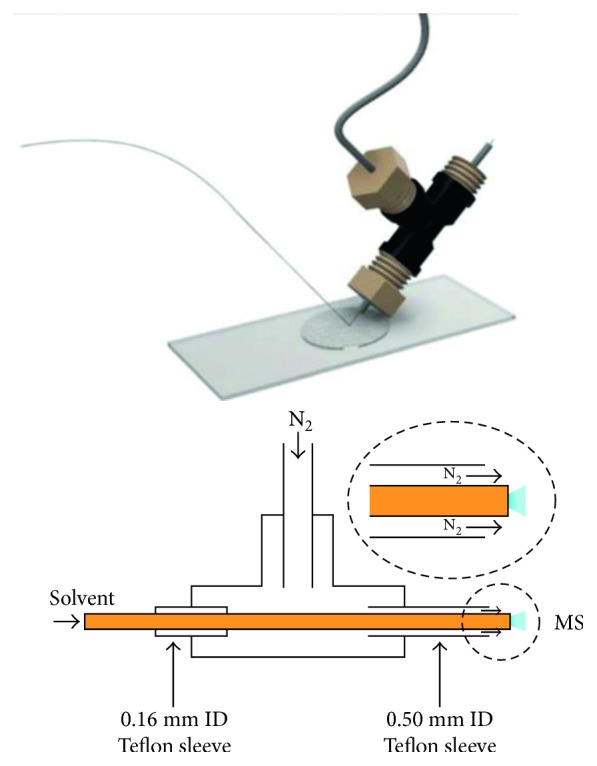
Picture and schematic representation of the pneumatically assisted nano-DESI ionization source. Reprinted with permission from [78].

A further instrumental innovation has been proposed by Duncan and coauthors [78], who developed a pneumatically assisted nanospray desorption electrospray ionization source. The instrumental setup was based on the introduction of a secondary nebulizer replacing the self-aspirating secondary capillary in order to assist solvent flow, to promote desolvation of the analyte, and to increase the distance between the nanospray and the MS inlet (Figure 5).

The developed device was tested for the analysis of rat kidney tissue sections, allowing us to obtain an improvement in sensitivity of about 1–3 orders of magnitude compared to the conventional setup. In addition, ion images characterized by high contrast, suitable for more intricate studies of metabolite distribution in biological samples, were obtained. A more complete desolvation of the analytes and reduced ionization suppression were additional features of the proposed device.

### 3.2. Extractive Electrospray Ionization-Mass Spectrometry and Laser Ablation Electrospray Ionization-Mass Spectrometry

Extractive electrospray ionization (EESI) has been introduced in 2006 by Chen et al. [79]. It is based on the use of two different sprayers: the ESI sprayer generates a charged solvent spray, whereas the sample sprayer has the function to nebulize the sample solution from an infusion pump. The analytes are ionized in the collision area of the two sprays, and then, they are collected by the MS inlet.

The ionization mechanism of the EESI ion source was studied by Wang et al. [80], and different MS-based methods for the analysis of organic aerosols [81], drugs [82], pesticides [83], amino acids [84], and biomarkers [85] in different matrices were developed in the recent years.

**Figure 6:**
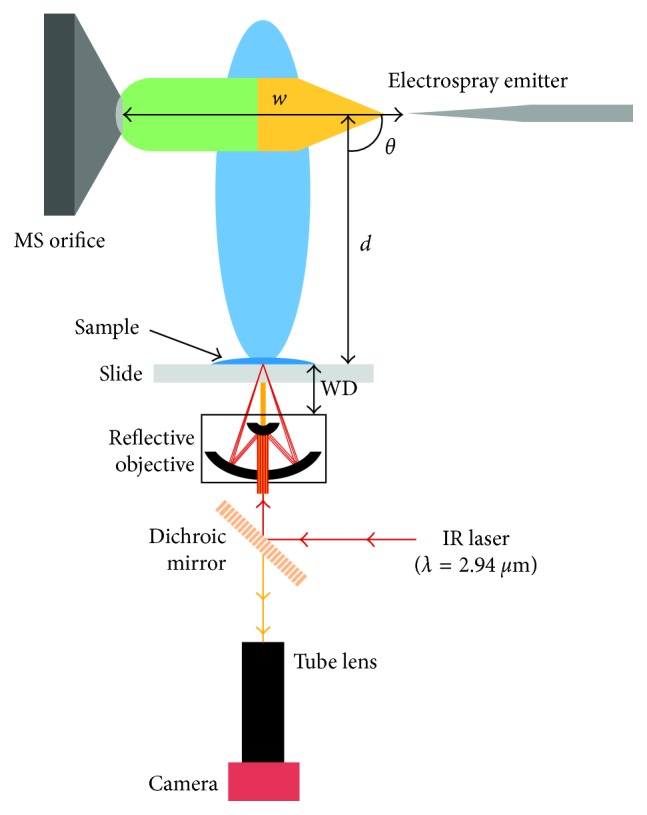
Schematic representation of tg-LAESI-MS. Reprinted with permission from [86].

LAESI-MS is another ambient ionization technique developed in 2007 by Nemes and Vertes [12]. Since most cells used for biomedical applications are cultured adherently, the use of LAESI-MS was proposed to analyze adherent cells directly onto the culture surface, thus avoiding chemical modification deriving from their detachment [86]. In order to reduce the LAESI spot size, the authors applied a transmission geometry- (tg-) LAESI and incorporated an objective with a high numerical aperture, thus achieving spot sizes in the 10–20 *μ*m range. This technique (Figure 6) was tested for the analysis of adherent versus suspended mammal cells, highlighting a difference in the metabolite compositions, thus proving that the cell detachment usually performed is able to produce chemical changes. On the contrary, tg-LAESI-MS allowed us to analyze directly the cells in their native state and, due to the smaller spot size, to reduce the sampled cell population by a factor of 20.

Optical microscopy combined with LAESI-MS has been suggested by Compton et al. [87] in order to acquire both morphological and chemical information from tissue sampling. In the developed instrumental setup, laser ablation occurred inside a chamber placed under an optical microscope: the ablated particulates generated by the laser were transported through a transfer tube by using nitrogen as carrier gas and finally ionized by the ESI spray.

In order to compare the performances of the developed prototype with those of the conventional LAESI-MS, plant tissues were analyzed. In comparison with conventional LAESI, the developed technique was characterized by reduced sensitivity and dynamic range; however, these features were still sufficient for the analysis and characterization of numerous metabolites and lipids in different spatial regions of biological tissues.

### 3.3. Paper Spray Ionization-Mass Spectrometry

In the last ten years, approaches based on the direct ionization from solid substrates, such as paper spray, probe-based spray, leaf spray, and tissue spray, strongly increased. All these techniques are characterized by the generation of an electrospray directly from a probe. The analytes in the samples can either be ionized directly upon the substrate surface or be extracted on a probe and subsequently ionized within few minutes, thus boosting analysis speed.

Innovations in PSI-MS have been described by Duarte et al. [88] and Salentijin et al. [89] who developed 3D-printed cartridges in order to obtain a solvent reservoir, thus allowing us to prolong the spray generation from the paper tip. A supporting prototype able to automatically perform PSI-MS analysis of a great number of samples, suitable for high-throughput applications, has been designed by Shen et al. [90]. Finally, the coupling of SFME with PSI-MS for the rapid analysis of macrolide antibiotics at the trace level in biological samples such as whole blood, milk, and other body fluids has been proposed in a study carried out by Deng et al. [91]. The same approach was applied for the detection of perfluorooctanesulfonic acid and perfluorooctanoic acid from *Daphnia magna* body fluids. After the SFME extraction, the organic extract was simply spotted on the PSI paper.

Excellent results were achieved in terms of linearity range (5–500 and 0.5–50 ng/ml range for antibiotics and perfluorocompounds, resp.) and sensitivity (LOQs 0.3–1.3 and 0.03–0.30 ng/ml for antibiotics and perfluorocompounds, resp.). Recovery rates always higher than 85% were obtained.

A novel paper spray cartridge with an integrated solid-phase extraction column has been developed by Zhang and Manicke [92]. The system was designed in order to perform on the same device the extraction, preconcentration, and ionization of the analytes from complex matrices such as blood or plasma. The cartridge was divided into two parts: the bottom one containing the absorbent waste pad and the paper spray substrate and the top one presenting a hole to host the solid-phase extraction (SPE) column. The procedure for performing paper spray analysis is the following: (i) the samples are loaded onto the SPE column (sample volume from 10 *μ*l up to hundreds of microliters); (ii) the unbounded compounds pass through the SPE column and are absorbed by the waste paper pad; (iii) after sliding the top part of the cartridge to the paper spray substrate, the analytes retained on the SPE column are eluted and analyzed by PSI-MS. The analytical performances in terms of detection and quantitation limits, recovery, and ionization suppression were evaluated for carbamazepine, atenolol, sulfamethazine, diazepam, and alprazolam. The SPE cartridge allowed both the selective enrichment of the targeted analytes from large sample volumes (up to hundreds of *µ*l) and the removal of interfering compounds, thus enhancing signal intensities. Compared to direct PSI, the proposed method allowed us to improve quantitation limits by a factor of 14–70, obtaining limits in the 0.2–7 ng/ml range.

### 3.4. Wooden-Tip Electrospray Ionization-Mass Spectrometry

Wooden-tip electrospray ionization-mass spectrometry (WT-ESI-MS) is a rapid, in situ, and direct ambient technique based on the use of a wooden tip as a sampling and ionization needle. The tip is dipped into the liquid solution/matrix, and after extraction, it is directly used as an ESI probe by applying a high voltage and spray solvent. The analytes enriched on the tip are desorbed and ionized under ambient and open-air conditions.

This method has been successfully applied by Yang et al. [93] for the analysis of pesticides, toxicants, date rape drugs, and illicit additives in various food samples. The capabilities of untargeted WT-ESI-MS analysis for the identification of the sources of plant materials by using a multivariate statistic approach were exploited by Xin et al. [94], whereas Yang and Deng [95] used an internal standard WT-ESI-MS-based method to obtain the fingerprint mass spectra for rapid quality assessment and control of Shuang-Huang-Lian oral fluid, an herbal preparation registered by Chinese Pharmacopoeia. By using the internal standard and principal component analysis (PCA), it was possible to obtain the fingerprints of samples from different manufacturers. A bamboo pen nib shaped and used for sample loading and an ESI probe for the determination of 4-chloro-amphetamine was developed by Chen et al. [96], resulting in lower detection limits compared to PSI-MS and traditional WT-ESI-MS analyses.

Similarly, a WT-ESI-MS method combined with different nonpolar solvents for the detection of native proteins and protein complexes directly from raw biological samples has been proposed by Hu and Yao [97].

The applicability of field-induced wooden-tip electrospray ionization-mass spectrometry (FI-WT-ESI-MS) for high-throughput analysis of herbal medicines has been proved by Yang et al. [98]. Field-induced ESI was performed by applying a high voltage on the MS inlet, thus allowing the creation of a strong electric field between the capillary emitter and MS inlet to induce ESI from the sample solution. A high-throughput analysis device was developed by the application of sample-contactless high voltage on the MS inlet. In addition, the switch between positive and negative ion detection modes can be readily accomplished, thus providing complete MS information of the analyzed samples. This approach allowed us to boost the analysis speed: 6 s per sample was sufficient to perform the analyses.

The proposed method was applied for the rapid determination of various active ingredients in different raw herbs and herbal medicines. The obtained mass spectra were used as fingerprints for tracing the origins, establishing the authenticity and assessing the quality of herbal medicines.

Very recently, a novel and noninvasive sampling method using a watercolor pen (brush) rinsed with ethanol as a sampling tool to collect analytes from the eyelids of volunteers has been evaluated [99]. The brush was placed between the mass inlet and the ESI plume, thus allowing the desorption and ionization of the analytes. The results achieved proved the suitability of the developed technique for the semiquantitative determination of caffeine and its metabolites in eyelid samples.

### 3.5. Miscellaneous

Following the method developed by Pan et al. [100], based on the use of a single probe inserted into a single cell for sampling intracellular compounds by real-time MS analysis, Chen et al. [52] described a novel method for single cell analysis and lipid profiling by combining drop-on-demand inkjet cell printing and probe electrospray ionization-mass spectrometry (PESI-MS). Droplets containing single cells were generated from a cell suspension by inkjet sampling, precisely dripped onto the tungsten tip of the ESI needle, and sprayed under a high-voltage electric field. Cellular lipid fingerprints were then obtained by MS detection. The analytes from eight types of cells were detected, and PCA analysis was performed in order to differentiate the samples. The proposed platform was demonstrated to be suitable for the direct MS profiling of single-cell lipid species without derivatization or the labeling procedure.

A method for the direct characterization of metals in solid samples using electrospray laser desorption ionization-mass spectrometry (ELDI-MS) has been developed by Shiea et al. [101]. The main advantages over classic approaches were related both to the absence of sample pretreatment and to the presence of very short analysis time. Mixtures of metal-EDTA complexes were applied on a stainless steel plate and submitted to ELDI-MS analysis. The capabilities of the technique were initially tested by spotting the metal-EDTA complexes on a solid probe and performing laser ablation of the material. The ablated analytes, present as EDTA complexes, were ionized in the electrospray plume. Further experiments were carried out by deposing the sample on the probe surface while maintaining the EDTA in the ESI spray solvent (functional electrospray). Excellent results in terms of sensitivity were achieved, thus proving method reliability for the rapid analysis of metal substrates without sample preparation.

An electrostatic spray ionization (ESTASI) method for the analysis of samples deposited in or onto an insulating substrate has been proposed by Qiao and coworkers [102]. In this study, the ionization of the analytes is induced by a capacitive contactless coupling between the electrode and the sample: by applying a pulse high voltage to the electrode, an electrostatic charging of the sample occurs, leading to a bipolar spray pulse. When the applied voltage is positive, the bipolar spray pulse consists first of cations, followed by anion production. The instrumental setup can be modified in order to obtain ion emission from samples in a silica capillary, in a disposable pipette tip, and in a polymer microchannel or deposited as droplets onto an insulating poly(methyl methacrylate) plate presenting wells or hydrophilic patches. This technique proved to be suitable for the analysis of peptides and proteins.

## 4. Materials for Spray-Based Ionization Techniques

The development of new materials is a field of increasing interest in order to enhance the performances of analytical methods. Both high selectivity and increased ionization efficiency are demanded to detect analytes at trace levels in complex matrices and to shorten analysis time. Different studies have been published dealing with the development of novel surfaces for ambient MS. A brief overview on the most recent materials developed for spray-based ionization techniques with particular attention to DESI-MS and PSI-MS applications is described in the next paragraphs.

### 4.1. Materials for DESI-MS

In 2005, Takats et al. [103] demonstrated that the DESI ion source is strongly influenced by the dielectric constants of both the substrate material and the spray.

The effect of surface chemistry in the DESI ionization mechanism has been deeply investigated by Penna and coworkers [104]: the performances of different glass substrates obtained by the sol-gel technology and functionalized by using different alkylsilanes were tested and compared in terms of ionization efficiency. The substrates were characterized in terms of surface free energy and wettability. Owing to their different polarity, melamine, tetracycline, and lincomycin were used as model compounds. A significant decrease in the ionization efficiency was observed when more hydrophilic surfaces were used, thus demonstrating the pivotal role of both hydrophobicity and wettability to increase the performances of DESI-MS experiments.

More recently, a 3D printed polylactic (PLA) supports in order to detect insulin and gentamicin in chitosan gels have been proposed by Elviri and coauthors [105]. By using 3D printing, hemispherical wells were created, thus allowing us to obtain DESI-MS responses five times higher than those achieved by using PTFE commercial slides. Improvement in terms of signal stability was also achieved, thus suggesting the capability of 3D printing technology to improve the desorption step in DESI-MS.

**Figure 7:**
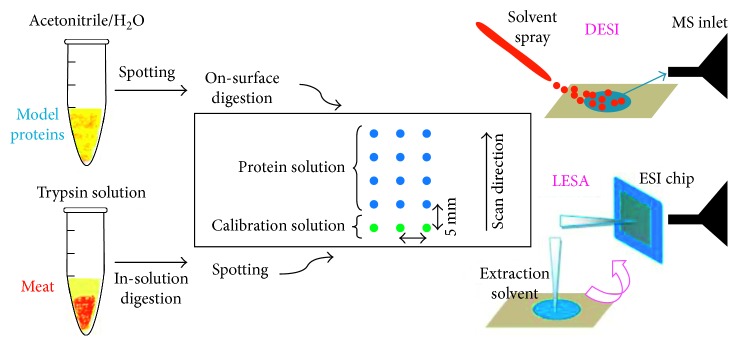
Schematic representation of the procedure proposed by Montowska et al. Reprinted with permission from [106].

Novel substrates have been proposed also for proteomics and peptidomics: the capability of Permanox slides for both DESI-MS and liquid extraction surface analysis-mass spectrometry (LESA-MS) of skeletal muscle proteins obtained from a mixture of standard proteins and raw meat has been discussed in a recent study [106]. The proposed method is schematically reported in Figure 7.

In both cases, good responses were obtained with LESA-MS characterized by higher sensitivity and stability with respect to the DESI-MS approach. Finally, multivariate data analysis allowed the correct discrimination among different meat classes.

Rapid and simple analyses represent a key parameter in proteomics: an interesting study was carried out by Dulay et al. in 2015 [107]. Two hybrid organic-inorganic organosiloxane polymers functionalized by immobilized trypsin (T-OSX) for the on-surface and in situ digestion of four model proteins, that is, melittin, cytochrome c, myoglobin, and bovine serum albumin, were developed and tested under DESI-MS and nano-DESI-MS conditions. The silica polymers were obtained via sol-gel technique using methyltrimethoxysilane and dimethyl-dimethoxysilane as precursors. The OSX polymers were derivatized with trimethoxysilylbutyraldehyde and functionalized with different amounts of trypsin. In both cases, despite the low enzyme-to-substrate ratios, the achieved results proved the suitability of the developed substrates to allow on-surface protein digestion followed by direct DESI-MS analyses obtaining sequence coverages in the 65–100% range.

Taking into account that DESI-MS analyses can be performed also on liquid samples, an improvement of the apparatus commonly used for this kind of analyses has been proposed [108] by replacing the sample transfer silica capillary with a trap column filled with chromatographic stationary phase materials, such as C_4_ and C_18_. The proposed system proved to be suitable for trace analyses of both organic compounds and biomolecules such as proteins/peptides in complex matrices in the presence of high salt content. Another interesting feature was related to the covalent functionalization of the inner wall of the sample transfer capillary with enzymes, thus allowing the fast and online digestion of proteins.

Noticeable applications of DESI-MS are based on its coupling with new sampling devices and extraction and separation techniques to develop methods for high-throughput analyses.

The microfabrication of ultrathin-layer chromatography (UTLC) plates via conformal low-pressure chemical vapor deposition of silicon nitride onto patterned carbon nanotube (CNT) scaffolds, acting as surface templates, has been described by Kanyal et al. [109]. The plates were heated and oxidized to both remove the CNTs and convert Si_3_N_4_ into silica; finally, the plates were hydroxylated in aqueous ammonium hydroxide. The resulting UTLC phases did not show any distortion of the microfeatures and were characterized by a higher robustness in comparison to high-pressure TLC plates. Good results in terms of chromatographic performances were observed obtaining a faster separation when mixtures of lipophilic, water-soluble, and fluorescent dyes are to be analyzed. A strong reduction in terms of mobile-phase consumption and an enhanced lifetime were observed. Finally, the UPTLC plates were submitted to both DESI-MSI and direct analysis in real-time (DART)-MS analyses, showing a good compatibility with common ambient desorption and ionization techniques.

In the same year, Ewing et al. [110] described the DESI-MS detection of the low vapor pressure nerve agent simulant triethyl phosphate. The analyte was previously adsorbed onto silica gel, forming a very fine particulate that was collected by using a sticky screen sampler. The device was characterized by a stainless steel screen presenting a partially polymerized polydimethylsiloxane (PDMS) coating. The quantitative collection of the particulate sample from a contaminated surface was achieved by interfacing the sticky screen sampler with a bioaerosol collector. Finally, the sticky screen was placed onto a moveable platform mounted in front of the DESI-MS instrument, thus allowing a reproducible sample introduction system able to minimize sampling errors. DESI-MS analyses performed directly on the PDMS coating allowed us to obtain a very low detection limit suitable for trace detection.

Electrospun nylon-6 nanofiber mats for DESI-MS analysis and imprint imaging have been developed by Hemalatha et al. [111]. The nanofibers were developed by needleless electrospinning: nylon-6 was dissolved in formic acid and the solution was electrospun at room temperature. Uniform mats of varying thicknesses composed of ∼200 nm diameter fibers were obtained: the properties of these materials can be tuned by varying spinning conditions and surface functionalization. As model compounds, dyes and the extract of periwinkle flower were spotted onto the nylon nanofiber mat, thus obtaining a uniform coating of the fibers. DESI-MS analysis allowed us to obtain spectra without polymer interference and reproducible DESI-MS images. The authors also demonstrated that compounds of interest could be incorporated into nanofibers during their formation by using as model compounds the crude methanol extract of periwinkle flower and tetraphenylphosphonium bromide (TPPB). The major metabolites of periwinkle were identified by DESI-MS, even though the spectrum was different compared to that obtained by spotting the sample. TPPB was detected with no nylon interference. The authors demonstrated the possibility of imprinting patterns made of printing inks, plant parts, and fungal growth on fruits on the nanofiber mats. Metabolites were identified by DESI-MS. The results highlighted that electrospun nanofiber mats could be considered as smart surfaces to capture diverse classes of compounds for rapid detection or to imprint imaging under ambient conditions.

### 4.2. Materials for PSI-MS

Although traditional paper spray ionization is performed on the filtering and chromatographic paper [11, 112–115], researchers have focused their attention to the development of new functionalized substrates in order to obtain substrates characterized by enhanced selectivity and sensitivity.

Very recently, Lai et al. [116] compared the ionization performances of different paper-like substrates obtained from both natural fibers and synthetic fibers, namely, gampi paper, Tengujou paper (natural), polycarbonate, polylactic acid, and poly-l-lactic acid (PLLA) (synthetic), with those of traditional chromatographic paper for the analysis of designer drugs. The surface characterization of the developed materials showed the presence of different surface morphologies able to affect PSI capabilities: gampi paper and PLLA nanofibers, characterized by a tough and extremely thin structure, were able to promote signal enhancement, thus allowing us to reach lower limits of detection. These findings could be explained by taking into account the reduced thickness of the used papers: by operating under the described conditions, a rapid evaporation of the sample molecules occurs, thus increasing the speed of the ionization process.

The analytical performances of paper with paraffin barrier (PS-PB) for the PSI-MS detection of hydroxymethylfurfural (HMF) and sugars like glucose and xylose in sugarcane liquors have been tested by Colletes et al. [117]. Microfluidic hydrophobic channels were obtained using paraffin barriers on paper substrates, thus delimiting a region for inducing the sample inlet into the mass spectrometer. The proposed stamping method allowed rapid prototyping of microfluidic paper-based analytical devices, without the need of sophisticated instrumentation. Different types of papers were investigated: an increase of the PSI-MS responses of xylose and glucose as a function of the decrease of porosity of the paper substrate was observed. PS-PB showed the best performance compared to the conventional paper and paper with two rounded corners. PS-PB was applied to detect sugars and their inhibitors in liquors from a second-generation ethanol process, thus obtaining excellent results in terms of linearity (over two orders of magnitude) and limits of detection and quantification.

Another interesting study carried out by Zhang et al. [118] proposed the use of commercially available silica-coated paper in order to increase the PSI-MS responses of therapeutic drugs in dried blood spots. The presence of silica gel particles in the cellulosic framework of the silica-coated paper produced a reduced diffusion of the blood through the substrate, thus leading to a higher percentage of blood sample available on the top side of the substrate. By operating under the optimized conditions, that is, by using dichloromethane/isopropanol (9 : 1 v/v) as a spray solvent mixture, limits of quantitation of about 0.1 ng/mL were achieved, with a sensitivity gain of 5–50-fold in comparison to chromatography papers.

CNT-coated filter paper for low-potential PSI-MS analysis of different organic molecules has been used by Narayanan and coauthors [119]: by applying a voltage of 3 V, full-range mass spectra similar to those obtained by conventional ESI at 3 kV could be recorded. The advantage of the proposed material relies on the use of very mild conditions for the ionization of the analytes, thus allowing the detection of compounds that could be easily oxidized. The performances of the proposed analytical method were assessed for a wide range of volatile and nonvolatile compounds, such as amino acids, antibiotics, and pesticides in different matrices.

Very recently, Wei et al. [120] synthesized graphene oxide (GO) nanosheet-modified N^+^-nylon membrane (GOM) for the extraction of malachite green (MG), a highly toxic disinfectant, and its metabolites in liquid samples and fish meat. GO nanosheets are characterized by a very high surface area (∼800 m^2^/g), suitable for MG adsorption via *π*-*π* stacking and electrostatic interactions. GOM was obtained by self-deposition of GO thin films onto N^+^-nylon membranes. The material was tested both as a direct spray ionization substrate and as an LDI-MS probe. The latter application resulted in no significant response, whereas the coupling between GOM and direct spray ionization allowed the quantitation of MG and its metabolites at nanomolar levels with extraction recoveries higher than 98%.

An improvement of the performances of PSI-MS in terms of sensitivity has also been achieved in a study dealing with the use of a paper substrate functionalized with urea [121]. Triangles of chromatography paper were treated with 1-[3-(trimethoxysilyl)propyl]urea to create an anion capture layer. The authors demonstrated that the urea-modified paper is an excellent substrate for PSI-MS since it is able to reduce ionization suppression caused by anions and highly polar compounds in the negative-ion MS mode. These findings are of pivotal importance for the analysis of biological samples like urine, blood, and plant extracts.

A selective substrate for PSI-MS, based on the use of molecularly imprinted polymers (MIPs), has been proposed by Pereira et al. [122]. More precisely, a membrane spray ionization method, combining MIP extraction and PSI-MS analysis, was developed and tested for the determination of diuron and 2,4-dichlorophenoxyacetic acid from apple, banana, and grape methanolic extracts. Being used as PSI substrates, MIPs were synthesized directly on a cellulose membrane: the bulk of the MIP was made by ethylene glycol dimethacrylate, using monuron and 2,4,5-trichlorophenoxyacetic acid as templating agents. After extraction, the MIP membranes were washed to remove matrix interferences and tested as PSI-MS substrates using methanol as a spray solvent. The use of these novel materials allowed us to obtain signal intensities of the targeted analytes far higher than those obtained by nonimprinted polymers with detection limits in the *µ*g/l range.

Bills and Manicke [123] developed a disposable paper spray cartridge containing a plasma fractionation membrane to perform on-cartridge plasma fractionation from whole blood samples. Three commercially available blood fractionation membranes, made of different materials, ranging from polymers to natural and synthetic fibers, that is, Vivid polysulfone membrane, NoviPlex plasma fractionation card, and CytoSep, were tested. Even though all the materials were capable of interacting with plasma samples with low levels of cell lysis, difficulties in terms of drug extraction were observed. In particular, Vivid polysulfone membrane and NoviPlex plasma fractionation card exhibited a high binding capability (over 30%) for all the tested drugs, whereas CytoSep showed a lower binding affinity (<17%) only for two out of five drugs. A drawback of the developed device was also related to the poor fractionation efficiency, as measured by the red blood cell content in the fractionated plasma. Quantitative analysis of plasma using PSI-MS provided results closed to those obtained by HPLC-MS without the need of offline extraction or chromatography separation.

A new zero volt-paper spray ionization (ZV-PSI) has been developed recently by Wleklinski et al. [124]: this approach is based on the generation of the electrospray by the action of the pneumatic force of the vacuum at the MS inlet. ZV-PSI analyses were performed over a large variety of samples, including tributylamine, cocaine, terabutylammonium iodide, 3,5-dinitrobenzoic acid, fludioxonil, and sodium tetraphenylborate. In comparison to classic PSI-MS, the achieved results showed a strong decrease of signal intensities for all the investigated analytes. Although the range of analytes useful for ZV-PSI-MS analysis resulted to be very similar to standard PSI-MS, differences in mass spectra were obtained. The observed behavior was related to the ionization mechanism of the proposed approach, which is strongly related to the effects of analyte surface activity. By using a Monte Carlo simulation, the mechanism regarding the formation of ions from initially uncharged droplets was also explained, thus allowing us to predict detection limits very closed to those observed experimentally and to calculate the relative surface activity of both positive and negative ions.

### 4.3. Materials for Wooden-Tip Electrospray Ionization-Mass Spectrometry

Surface-coated wooden-tip electrospray ionization-mass spectrometry (SCWT-ESI-MS) is a new technique based on the use of a functionalized wooden needle, acting both as extraction/enrichment phase and ESI probe. By using this strategy, the tip is coated by a proper sorbent for highly selective enrichment of targeted compounds from complex matrices, thus making it suitable for analyses at ultratrace levels. Luan suggested the use of a SCWT-ESI-MS technique to detect different analytes in complex matrices [125, 126].

The SCWT-ESI-MS technique has been applied for the detection of perfluorinated compounds (PFCs) in complex environmental and biological samples at ultratrace level [125]. Sharp wooden tips were functionalized via the silanization process by using octadecyltrimethoxysilane and *n*-octadecyldimethyl[3-(trimethoxysilyl)propyl]ammonium chloride (OTPAC), in order to obtain two different extractive phases, characterized by long alkyl chain. The two phases were tested for the extraction of PFCs spiked water. The OTPAC coating was characterized by the best enrichment capabilities: the extraction is performed not only by the reversed phase, but also by exploiting the ion exchanged adsorption mechanism. Morphological studies of the tip showed a high probe porosity, thus increasing the surface area of the material, and presence of microchannels for transport of the solvent to achieve ambient ionization MS analysis. After method optimization, the probe was tested for the detection of eight different PCFs both in pure water and in complex matrices, that is, lake water, river water, whole blood, and milk. The achieved results proved that the SCWT probe is characterized by outstanding enrichment capabilities, thus being able to enhance method sensitivity by approximately 4000–8000-folds and 100–500-folds in aqueous samples and in whole blood and milk samples, respectively. Method validation resulted in good linearity (two orders of magnitude), excellent quantitation limits (in the 0.21–1.98 ng/l range), and accuracy (recovery rates in the 89–112% range).

Similarly, a SCWT-ESI-MS-based method has been tested for the rapid and sensitive analysis of trace fluoroquinolone and macrolide antibiotics in water [126]. The wooden probe was functionalized via silanization and sulfonation reactions in order to obtain a sulfo-C_8_-chain coating able to interact with the analytes with both reversed phase and ion exchange mechanisms. The SCWT-ESI-MS method was then optimized and tested for the extraction of four fluoroquinolone and two macrolide antibiotics in water at trace levels. Method sensitivity allowed us to obtain detection and quantitation limits in the 1.8–4.5 and 5.9–15.1 ng/l range, respectively. Again, linearity was verified over two orders of magnitude: good precision (RSD <14.3%) and recovery rates in the 93.6–112.6% range were other features of the developed method. Finally, the developed method was applied for the analysis of the targeted antibiotics in tap and river water samples.

**Figure 8:**
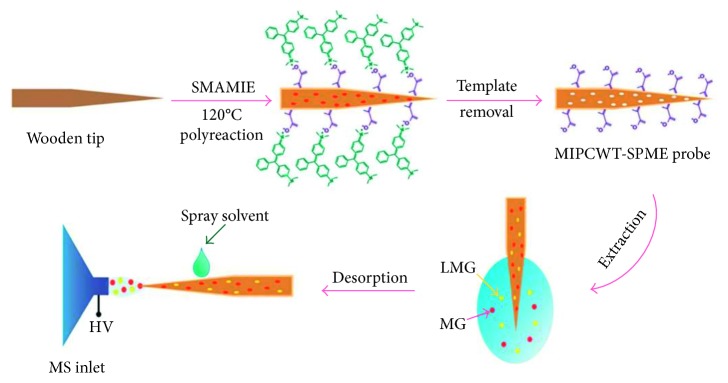
Schematic representation of the instrumental setup proposed by Huang. Reprinted with permission from [127].

An interesting approach based on the use of molecularly imprinted polymers as coating for SCWT-ESI-MS has been discussed in a recent study [127]. The coating was synthesized by applying a silicone-modified acrylate molecularly imprinted emulsion onto the tip surface (Figure 8). The developed material was tested for the detection at trace levels of malachite green and its metabolite leucomalachite green in aqueous samples. The MIP-SCWT probe exhibited high enrichment capabilities, allowing us to obtain detection limits at low µg/l levels. In addition, a good linearity was obtained for both the compounds (three orders of magnitude). The method proved to be suitable for high-throughput analysis and was successfully applied for the analysis of tap water, river water, and fish samples.

### 4.4. Miscellaneous

Similar to PSI, aluminum-foil mass spectrometry (Al-ESI-MS) was recently developed by Hu et al. [128]. This technique is based on the use of a household aluminum foil to obtain the spray ionization of the analytes. The Al foil was cut into triangles, which were folded symmetrically to obtain a mini-reservoir for the sample solution, and was connected to the high voltage supply of the mass spectrometer. The proposed technique was tested for the direct analysis of a wide variety of complex matrices, namely, energetic beverages, urine, skincare and medical creams, and herbal medicines. The inert, hydrophobic and impermeable surface of the Al foil allowed effective on-target extraction of solid samples and on-target sample clean-up, that is, removal of salts, adulterants, and detergents from proteins and peptides. Being Al an excellent heat conductor, the direct monitoring of thermal reactions, such as thermal denaturation of proteins, can be performed in an easy way by Al-ESI-MS.

In a different research study, ESTASI was applied to identify and quantify different compounds from silica gel surfaces, via direct coupling with TLC [129]. The sample spots separated by TLC were analyzed by ESTASI-MSI. The analyses were performed on both drug molecules, using normal phase TLC, and dyes using reversed phase C18 TLC plates, thus guaranteeing the analyses of compounds characterized by very different polarity. After sample separation, the hydrophobic substrate was coated with chlorotrimethylsilane to form hydrophobic surfaces, suitable for ESTASI analysis. Both TLC plates were considered ideal substrates for in situ characterization of samples by using ESTASI-MS, with efficient analyte extraction and separation. In addition, the capability of removing interfering compounds such as salts increased method performance, thus allowing the detection of the investigated analytes at trace levels.

## 5. MS-Based Approaches for Food Analysis

The demand for safe and high-quality foods has significantly increased in recent years. Food safety and quality have become of greater importance, and the governments of many countries have increased the amount of relevant legislation and demands for food authentication [130]. In consequence, the development of more robust, efficient, cost-effective, and powerful analytical methodologies is continuously needed in order to face these requirements. MS is one of the most suitable techniques because it is featured by excellent specificity, sensitivity, and throughput [131]. MS has been widely used in food safety and quality analysis, and recent advances in MS can provide faster and more accurate methods able to offer better qualitative and quantitative results. Additionally, coupling mass analyzers with separation techniques, such as liquid chromatography (LC-MS) and gas chromatography (GC-MS), have significantly improved food analysis for screening, identification, structural characterization, and quantitation purposes.

One of the most challenges in the application of MS in food analysis, especially in detection of contaminants, is sample preparation because foods are considered very complex matrices in which some natural components can negatively influence the analysis of the targeted compounds. Traditional methods for sample extraction include solid-liquid extraction (SLE), liquid-liquid extraction (LLE), and solid-phase extraction (SPE). More recent is the use of solid-phase microextraction (SPME), pressurized liquid extraction (PLE), and QuEChERS (quick easy cheap effective rugged safe) [132]. The introduction of high-resolution mass spectrometers, which provide extremely high selectivity and sensitivity, or other emerging MS strategies such as ambient-ionization MS, direct food analysis, and matrix-assisted lased desorption ionization-time of flight-mass spectrometry (MALDI-TOF-MS) profiling and imaging, has strongly reduced sample preprocessing.

According to the PubMed database, since 2012 about 20000 publications dealing with developed MS-based methods for food safety and quality purposes are available. In this section, we do not intend to provide an exhaustive revision of all published studies, but an overview of the most important MS techniques proposed to evaluate food safety and quality.

### 5.1. MS-Based Approaches for Food-Safety Assessment

The main purpose of food analysis is to ensure food safety, thus requiring the development of accurate and reliable methodologies for the detection of microbial-related spoilage, determination of allergens, detection of environmental contaminants as well as banned external compounds, or the assessment of the occurrence of natural toxins. These methods are strongly influenced by current legislation, which establishes the requirements that an analytical method must meet for an unequivocal identification and quantification of a controlled substance in food samples, as well as the maximum residue limits (MRLs) on certain substances [133].

Being able to allow the quantification of known compounds with great selectivity and sensitivity, tandem MS detection is one of the most frequently utilized analytical approaches to determine contaminants in foods. Triple quadrupole (QqQ) mass spectrometers, running under multiple reaction monitoring (MRM) mode, are the most popular instruments for detecting contaminants in food. This detection procedure allows us to verify the compliance with European legislation on banned and controlled substances in foods [133].

Since 2010, numerous researches have used this methodology to detect pesticides in several fruits and vegetables, such as tomato [134–137], orange [138], mandarin [139], rice and red pepper [139–141], avocado [142, 143], apples and cucumbers [144, 145], mango [146], tea [147], lettuce [148], grains and cereals [149–152], soybean products [153], groundnut oil [154], and wines [155]. The same methodology was also used in the identification and quantification of veterinary drug residues in shellfish [156], meat [157, 158], eggs and milk [158], and contaminants from food contact materials [159].

Triple quadrupoles in MRM mode is also the most-extended approach to detect and quantify toxins and pathogens in food. These pathogens can contaminate foods directly or indirectly, through the productions of toxins. The control of toxin and pathogen levels is extremely important, since the consequences on health due to their contamination of food may be very serious. Following this approach, food products such as nuts [160], maize [161], shellfish [162], tomato [163], beer [164], and multicereal baby food [165] were analyzed.

Almost all these applications combine QqQ-MS with LC or GC separation methods. In some cases, LC- and GC-based techniques were also coupled with other types of MS analyzers such as ion traps (ITs) or quadrupole-linear ion traps (Q-LITs), TOF, or Q-TOF to determine food contaminants [166–168].

Multidimensional procedures allowed us to increase resolving power and separation capabilities that can be beneficial for subsequent MS-based detection, considering that the targeted compounds can reach the detector more separated in time. This is the case of comprehensive two-dimensional gas chromatography (GC × GC) that has been coupled to a TOF-MS analyzer to determine dioxin-related pollutants in complex food samples [169], to screen 68 pesticide residues in oilseed [170], or to detect and to quantify different polychlorobiphenyls (PCBs), polybrominated diphenyl ethers (PBDEs), and PAHs in fish samples [171].

More recently, HRMS analyzers, typically Q-TOF, Orbitrap-MS, and Fourier-transform ion cyclotron resonance- (FT-ICR-) MS, have been used in the field of food safety. These instruments are characterized by high resolution (100,000–1,000,000 FWHM) and high mass precision (1-2 ppm, allowing discrimination between isobaric interferences and ions of interest), thus making possible the screening of unknown compounds with a full MS scan and the construction of databases for targeted compounds. For instance, UPLC-Orbitrap-MS was used to create a database of more than 350 compounds in honey [172]. These databases included different classes of pesticides and veterinary drugs and allowed simultaneous screening of analytes and identification and quantitation of detected compounds in different honey samples. Similar UPLC-HRMS approaches have been lately used to create an accurate-mass database including the fragmentation of more than 600 different food contaminants, such as pesticides, veterinary drug residues, mycotoxins, and perfluoroalkyl substances [173]. Since the particle size of the stationary phase in UPLC is significantly lower than that observed in HPLC, UPLC yields higher speed, better resolution, increased sensitivity, and better peak capacity. Additional examples are related to the development of LC-Orbitrap-MS-based methods for pesticide screening in vegetables and fruits [174], as well as for the analysis of 18 selected mycotoxins in baby food [175].

Ambient MS-based techniques have also been widely applied for food safety purposes: different ionization techniques have been used like LAESI-MS to detect neurotoxin domoic acid in shellfish [176], DESI-MS for the rapid detection of shellfish poisoning toxins in mussels [39], and PSI-MS for the determination of pesticides in fruits and vegetables [114].

### 5.2. MS-Based Approaches for Food Quality Assessment

Besides food safety, food quality is one of the main concerns of the modern food industry. Food quality encompasses multiple factors, since food authentication and adulteration of food characteristics include food ingredients, such as lipids, proteins, oligosaccharides, vitamins, and carbohydrates, and additives, such as preservatives, antioxidants, and chemicals used for flavor, color, and odor. As a consequence, the evaluation of food quality usually represents a very complex task that needs to consider multiple aspects to achieve the appropriate food quality. Food composition, aroma, flavor, or nutritional properties are among the most important features that need to be evaluated in food quality assessments.

Several MS-based approaches have been used to determine food quality. The most recent publications have mainly used nontargeted MS-based approaches, which very often included the coupling LC-MS and/or GC-MS.

Among LC-MS analytical methods, LC-HRMS techniques have been used for quality evaluation of raw turmeric form different regions [177], for the discrimination of grapes according to plant sterol content [178], for the analysis of the metabolome of the Graciano *Vitis vinifera* wine variety [179], and for the investigation of the quality and authenticity of saffron [180] and strawberries . Moreover, methods based on the UPLC combined with HRMS were developed to assess the authentication and the evaluation of possible adulterations in saffron [181] and fruits juices [182, 183]. The last two methods rely on the feasibility of the application of the UPLC-QToF platform to perform both nontargeted and targeted methods to select potential biomarkers, which should make it possible to develop a targeted method (less sophisticated instrumentation and simpler data analysis) for routine analysis. Similarly, the combination of nontargeted and targeted methods was reported for the qualitative analysis of curcuminoids in turmeric [184]. In this case, nontargeted analysis was performed by using LC-QTOF-MS/MS and the targeted approach by LC-QTRAP-MS/MS.

In the LC-MS-based approaches devoted to food quality assessment, it is noteworthy to highlight the use of hydrophilic interaction chromatography (HILIC), especially in metabolomics approaches. HILIC columns allow profiling highly polar and hydrophilic compounds providing complementary metabolic information to reversed-phase LC. In spite of several caveats associated to HILIC, such as variability in retention times, low peak efficiency, and long re-equilibration times after gradient elution, this methodology has been successfully used to analyze contamination and degradation of infant formulas [185], to separate and detect marine toxins [186], or to identify biomarkers of meat quality [187].

GC-MS-based approaches have also been widely used to evaluate food quality. In these approaches, GC was coupled to a huge diversity of mass analyzers: from simple MS instruments, like quadrupole (Q) [188–192], IT [193], to high-resolution instruments [194–198], as well as hybrid analyzers [199–201]. Studies of the effect of volatile compounds for the classification of saffron based on the concentration of biomarkers [188], classification of olive oils on the basis of their quality parameters [200], the establishment of differences between wine grape cultivars [194], or the detection of milk or meat adulteration [78, 190] are only some of examples relying on the use of GC-MS platforms in food quality analysis.

Besides the much more common LC-MS and GC-MS platforms to assess food quality, comprehensive two-dimensional GC [202] and CE methods [186] coupled to TOF analyzers have also been applied. GC × GC allowed the creation of a panel of biomarkers of rice flavor quality through establishing associations between volatile metabolites and perception of rice aroma [202]. These results are valuable for breeding programs since they can be used to choose pleasant rice aromas. In the latter, the feasibility of using a polymer-coated-capillary for the separation of anionic metabolites in both orange juice and wine has been demonstrated [186]. It offers a complementary coverage of the metabolome of these samples to those provided by other analytical techniques.

In addition to spray-based ionization techniques [203–206], mass spectrometry imaging (MSI) is another useful technique for food safety and quality control through monitoring the spatial distribution of bioactive components and contaminants in food samples. Until recently, MSI was largely performed with MALDI. MALDI-TOF-MSI was successfully applied to investigate the distribution of toxic glycoalkaloids in potato tubers [207], to identify the site of capsaicin in *Capsicum* fruits [208], and to observe both the tissue site of 10 anthocyanin species in blueberries [209] and the posttranslational modified sites in the alpha-melanocyte-stimulatin hormone for carp and goldfish pituitary tissue, as well as their ratio change under different environmental conditions [210]. Although MALDI-MSI can detect compounds in a tissue section without extraction, purification, separation, or labeling, the slow speed of the analysis and the need for matrix deposition in MALDI-MS are critical disadvantages in food imaging applications because they involve analyte diffusion able to affect the original molecular distribution. The development of various ambient ionization techniques revived interest in MSI because of the direct surface sampling in front of a mass spectrometer with submillimeter resolution and no sample preparation. These techniques permit rapid, direct measurement of compounds present on the condensed sample phase and have become potential analytical tools for direct profile-imaging analysis in an atmospheric pressure environment, thus being particularly useful for food-quality control purposes.

Although the application of these techniques in food analysis is not yet fully established, some examples can be found in literature. As an example, ELDI-MS was applied to obtain the molecular profiling and spatial distribution of particular active components in two edible fungi species [211] as well as alpha-solanine and alpha-chaconine in potato [212].

DESI-MSI was used to reveal the spatial distribution of chlorogenic acids and sucrose across the bean endosperm [213], as well as the spatial and temporal distribution of rohitukine and related compounds during various stages of seed development [214]. LAESI allowed macroscopic and microscopic imaging of pesticides, mycotoxins, and plant metabolites in different matrices [215].

## 6. Conclusions

MS-based techniques represent a highly valuable tool for environmental, bioanalytical, food safety, and food-quality control purposes and their application in these fields strongly increased in the past years. Despite the numerous advantages of MS-based methods, one of the most challenging aspects is still related to the analysis of complex matrices for the detection of nontarget or unknown compounds. The creation of detailed libraries of compounds, including MS-based information such as accurate mass, isotopic patterns, and collision-induced fragmentation, is strongly demanded.

Innovations in ambient MS allowed the development of analytical methods characterized by high-throughput and minimal sample preparation, thus allowing the analysis of samples in their native ambient. However, an important feature to be discussed when ambient MS methods are used is related to the concentration of the detected compounds on the sample surface that might not represent the actual concentration in the whole sample, thus not matching the requirements of current legislation or official methods of analysis.

## Nomenclature

CE: Capillary electrophoresis
CNT: Carbon nanotube
DART: Direct analysis in real time
DESI: Desorption electrospray ionization
DMPA: *N*,*N*-dimethyl-*p*-phenylenediamine
EASI: Easy ambient sonic-spray ionization
EC: Electrochemical
EESI: Extractive electrospray ionization
EI: Electron ionization
ELDI: Electrospray laser desorption ionization
ESI: Electrospray ionization
ESTASI: Electrostatic spray ionization
FA: Fatty acid
FFA: Free fatty acid
FI: Field-induced
FT-ICR: Fourier-transform ion cyclotron resonance
GC: Gas chromatography
GC × GC: Two-dimensional gas chromatography
GO: Graphene oxide
GOM: Graphene oxide membrane
HMF: Hydroxymethylfurfural
HILIC: Hydrophilic interaction liquid chromatography
HPLC: High-performance liquid chromatography
HRMS: High-resolution mass spectrometry
LIT: Linear ion trap
IT: Ion trap
LAESI: Laser ablation electrospray ionization
LC: Liquid chromatography
LEI: Liquid-electron ionization
LESA: Liquid extraction surface analysis
LLE: Liquid-liquid extraction
LS: Liquid sample
LOQ: Limit of quantitation
MALDI: Matrix-assisted laser desorption/ionization
ME: Matrix effect
MG: Malachite green
MIP: Molecularly imprinted polymer
MRL: Maximum residue limit
MRM: Multiple reaction monitoring
MS: Mass spectrometry
MSI: Mass spectrometry imaging
MS/MS: Tandem mass spectrometry
OTPAC: Octadecyldimethyl[3-(trimethoxysilyl)propyl]ammonium chloride
PAHs: Polycyclic aromatic hydrocarbons
PB: Paraffin barrier
PBDE: Polybrominated diphenyl ether
PCB: Polychlorobiphenyl
PDMS: Polydimethylsiloxane
PESI: Probe electrospray ionization
PFC: Perfluorinated compound
PMMA: Poly(methyl methacrylate)
PLA: Polylactate
PLE: Pressurized liquid extraction
PLLA: Poly-l-lactic acid
PSI: Paper spray ionization
PTFE: Polytetrafluoroethylene
Q: Quadrupole
QqQ: Triple quadrupole
QuEChERS: Quick easy cheap effective rugged safe
RSD: Relative standard deviation
SCWT: Surface-coated wooden tip
SFME: Slug-flow microextraction
SLE: Solid-liquid extraction
SMB: Supersonic molecular beam
SPE: Solid-phase extraction
SPME: Solid-phase microextraction
TLC: Thin-layer chromatography
TOF: Time of flight
TPPB: Tetraphenylphosphonium bromide
UPLC: Ultraperformance liquid chromatography
UTLC: Ultrathin-layer chromatography
WT: Wooden tip
ZV-PSI: Zero volt-paper spray ionization.
